# Texture Synthesis Repair of RealSense D435i Depth Images with Object-Oriented RGB Image Segmentation

**DOI:** 10.3390/s20236725

**Published:** 2020-11-24

**Authors:** Longyu Zhang, Hao Xia, Yanyou Qiao

**Affiliations:** 1 Aerospace Information Research Institute, Chinese Academy of Sciences, No.9 Dengzhuang South Road, Haidian District, Beijing 100094, China; zhangly@aircas.ac.cn (L.Z.); qiaoyy@aircas.ac.cn (Y.Q.); 2 Aerospace Information Research Institute, University of Chinese Academy of Sciences, No.19(A) Yuquan Road, Shijingshan District, Beijing 100049, China

**Keywords:** depth image, texture synthesis, object-oriented image segmentation, sample block, hole filling

## Abstract

A depth camera is a kind of sensor that can directly collect distance information between an object and the camera. The RealSense D435i is a low-cost depth camera that is currently in widespread use. When collecting data, an RGB image and a depth image are acquired simultaneously. The quality of the RGB image is good, whereas the depth image typically has many holes. In a lot of applications using depth images, these holes can lead to serious problems. In this study, a repair method of depth images was proposed. The depth image is repaired using the texture synthesis algorithm with the RGB image, which is segmented through a multi-scale object-oriented method. The object difference parameter is added to the process of selecting the best sample block. In contrast with previous methods, the experimental results show that the proposed method avoids the error filling of holes, the edge of the filled holes is consistent with the edge of RGB images, and the repair accuracy is better. The root mean square error, peak signal-to-noise ratio, and structural similarity index measure from the repaired depth images and ground-truth image were better than those obtained by two other methods. We believe that the repair of the depth image can improve the effects of depth image applications.

## 1. Introduction

There are many real-world scene data capture methods used in the computer vision and geographic information fields. A traditional visual camera only supports the capture of two-dimensional (2D) RGB images. Binocular Stereo Vision and light detection and ranging (LiDAR) were commonly used to obtain three-dimensional (3D) spatial information. However, the robustness of binocular vision is limited and LiDAR equipment is not power efficient. Moreover, LiDAR is not simple to use on portable devices. Thus, a depth camera is a good alternative to binocular vision and LiDAR in collecting 3D information. A depth camera can directly capture the distance between an object and the camera. Owing to the initial high price of the equipment, there were very few users of early depth cameras, and the related research was also sparse. However, since the launch of Kinect’s low-price depth cameras in 2011 [1], the number of users of low-price depth cameras has gradually increased, and the application of depth cameras has become increasingly common. The combination of depth and RGB images can be used in action recognition [2,3,4], simultaneous localization and mapping (SLAM) [5,6,7], 3D reconstruction [8,9,10,11], augmented reality [12,13,14] and other geographic information applications. The resolution of a depth image collected by the early Kinect depth sensor was only 640 × 480 pixels; hence, the data were not ideal. The device was competent for a gesture recognition task [15,16,17], but could not meet the needs of other services. Accordingly, various manufacturers have since launched different depth camera hardware products, such as Ailook [18], Astra [19], and Intel RealSense [20]. In the RealSense family of depth cameras, the model D435i is the latest and most popular product. The Intel RealSense depth camera D435i [21] has comparatively good characteristics, especially in terms of image resolution and frames per second; as well as overall device size, weight, and price.

The depth sensor directly obtains distance and scale information optically. Compared with other devices, the depth sensor has its own advantages. The working methods of depth cameras were mainly based on the use of structured light and time of flight (TOF). They are two active depth data acquisition methods. The RealSense D435i depth sensor used in this study is based on structured light. It is equipped with both left and right infrared cameras to collect depth data. The left and right infrared receivers are used to receive the infrared light; infrared dot matrix projectors are present in the middle of the device, which can enhance the exposure of the infrared band. In an indoor environment, the projectors can significantly improve the image quality of the infrared image and improve the accuracy of the depth image. The right-most RGB camera is used to collect visible-light signals. An active stereo sensor produces noise owing to the non-overlapping of image areas or lack of texture [22]; the presence of system noises also produces noises in the form of holes in the captured depth images. The quality of captured depth images is poor because of the holes; hence, the images cannot be directly used in practical applications, such as 3D reconstruction. To use depth data, which can directly provide scale information in augmented reality, automatic driving, visual positioning, and other geographic information applications, it is necessary to improve the quality of the depth data collected by the RealSense D435i so that it can provide the accuracy required by practical applications.

In early image inpainting works, there are lots of works that have reference significance for depth image inpainting. Bertalmio et al. [23] proposed a repair algorithm, named BSCB (Bertalmio Sapiro Caselles Ballester), which takes the vertical direction of the image damaged edge contour as the repair direction. On the basis of the BSCB algorithm and total variation denoising algorithm, Chan and Shen [24] proposed a TV (total variation) algorithm. The algorithm has a good image repair effect, but the repairing time is long. He et al. [25] added two threshold parameters to the TV model and improved the weight coefficient in the original algorithm. The algorithm not only ensures the repair effect, but also improves the repair speed. Gunnewiek et al. [26] proposed a method to automatically detect and fill the blocked area using the time axis. They start by analyzing the data along the time axis and calculate the confidence level for each pixel. Then, according to the calculated confidence level, the invisible pixels from the future and the past in the current frame are weighted and accumulated. At present, there are mainly four kinds of depth image repairing methods. The first method is a depth image filtering algorithm based on a joint bilateral filter [27,28]. This kind of method can satisfactorily repair small area holes at the edge of objects; however, it will cause a certain degree of information loss owing to the filtering of the global area of the image. For large-area holes in depth images, the filter algorithm requires amount of iterations, a long repair time, it produces blurred depth images, poor visuals, and a has poor 3D model after reconstruction. The second method is depth image repairing after 3D reconstruction. For the Kinect depth sensor, which was very popular in the early years, KinectFusion is generally used for the hole filling of captured depth images [29]. KinectFusion uses RGB-depth (RGB-D) images to reconstruct 3D images and form a spatial point cloud. The interpolation result is mapped to the 2D image and converted to grayscale. Then, the grayscale image is linearly transformed and the grayscale domain is stretched within the set depth area. Subsequently, the image information point coordinates are transformed to repair the hole in the depth image. This algorithm is only suitable for inpainting images of rigid objects, not for non-rigid objects; moreover, it easily produces small-area error repair. The third method is based on RGB image clustering [30]. This method uses the clustering results of RGB images as the guide, searches the non-empty points of the guided area at the empty points, and selects the depth value of the points to fill in the holes. This method is superior to the joint bilateral filtering method, but the robustness of the algorithm is not satisfactory due to its dependence on clustering results. The last method involves image inpainting algorithms based on texture synthesis. These algorithms can be more reliable in repairing large broken area image. Among them, there are three classical texture synthesis algorithms: (1) nonparametric sampling texture synthesis algorithm proposed by Efos and Leung [31]; (2) real-time texture synthesis algorithm based on block sampling proposed by Liang et al. [32] and; (3) Criminisi algorithm [33]. The texture synthesis algorithm, a superior digital image inpainting algorithm, was proposed by Criminisi et al. It is a sample image inpainting method based on search matching, which had a good effect on the processing of large-area holes, but jagged edges easily occurred at the junction of the repaired area and the original region, which caused visual incongruity. The block size of this method is immutable, and the default size is 9 × 9. Zhang et al. [34] proposed an adaptive selection method of the sample block size by extracting the relevant texture features of the image. By determining the relationship between the relevant texture features and the optimal sample block size, the automatic selection of the sample block size was realized, and the ideal texture synthesis effect was obtained. Cao et al. [35] optimized the aforementioned methods and proposed an image inpainting algorithm based on adaptive sample block local search (ASB-LS). This method uses the average correlation of the structure tensor to adaptively select the size of the sample block. At the same time, the local search strategy is used to improve the matching efficiency; this effectively avoids the problems of image structure error propagation and blind search of matching blocks. Compared with the Criminisi method, the effect of hole repair was improved. In this study, the texture-based hole-filling method is adopted, which will not affect the known information of the image and fill large holes. We propose a new method using RGB information. The repair accuracy is better than the Criminisi and ASB-LS methods.

Besides the aforementioned works, several scholars have published their own research in the field of depth image restoration in recent years. Liu and Cao [36] proposed a depth image inpainting algorithm based on image fusion. The improved watershed algorithm was used to extract the edge information of the color image based on a KD-tree. After fusing the depth information of the nearest neighbor image with the depth information of the image, the depth information of each image was extracted and classified. Xu and Sun [37] proposed a sample image restoration method based on search matching, which has a good effect on processing large-area holes. However, in repairing depth image holes, the repaired area contacted with the original area first, which was likely to cause jagged edges, producing visual discordance. If the depth image was repaired in this way, then the 3D model based on this method would have jagged faults at the edge. Yao et al. [38] proposed to use an RGB image to repair holes in a depth image. Owing to the use of information in the RGB image, especially the texture information related to the depth image, the filling inaccuracy problem caused by the lack of information was improved. However, a mosaic effect easily appeared in the repaired area. Chang et al. [39] proposed hole filling based on texture similarity, depth enhancement based on texture similarity, and depth refinement by a rotation consultant to enhance the depth map. The proposed depth enhancement system can simultaneously suppress noise, fill holes, and sharpen target edges. Yang et al. [40] implemented a hole-filling method in the depth image of the Kinect sensor. First, the depth hole was marked as having eight priorities. Each depth hole was filled according to the depth contribution value of adjacent pixels. Then, the hole-filling results are combined with bilateral filtering. This method reduced false filling caused by false-color depth image fusion. Fu and Wu [41] designed and improved the fast-joint bilateral filtering algorithm to fill the missing data in a large cavity. Feng et al. [42] proposed a hole-filling method based on the adaptive background offset depth map. The combination of color similarity and background bias painting can adaptively fill depth map holes for different types of Kinect depth holes, which significantly improved the quality of Kinect depth maps. Cho et al. [43] proposed a depth image hole-filling method that converges two boundary pixels to the target boundary. They introduced a simple way to fill the gap between two edge ends. This method can solve the problem wherein one or more edge pixels exist between two boundary pixels. In the process of hole filling, the filling direction was changed repeatedly, alternating between horizontal and vertical direction until no hole was left. Compared with the Criminisi method, this method is better.

In the present study, we use a texture synthesis algorithm to repair the depth image of RealSense. Moreover, we use an RGB image to perform multi-scale object-oriented image segmentation and add object constraints to the process of selecting the best sample block for depth image restoration to increase repair accuracy.

## 2. Methodology

### 2.1. Block Priority Calculation

We use the priority mechanism from the Criminisi method [33] to calculate block priorities of depth images, which realizes that the depth image can be repaired in a certain order, and the edge texture of the image can be repaired first.

**Figure 1:**
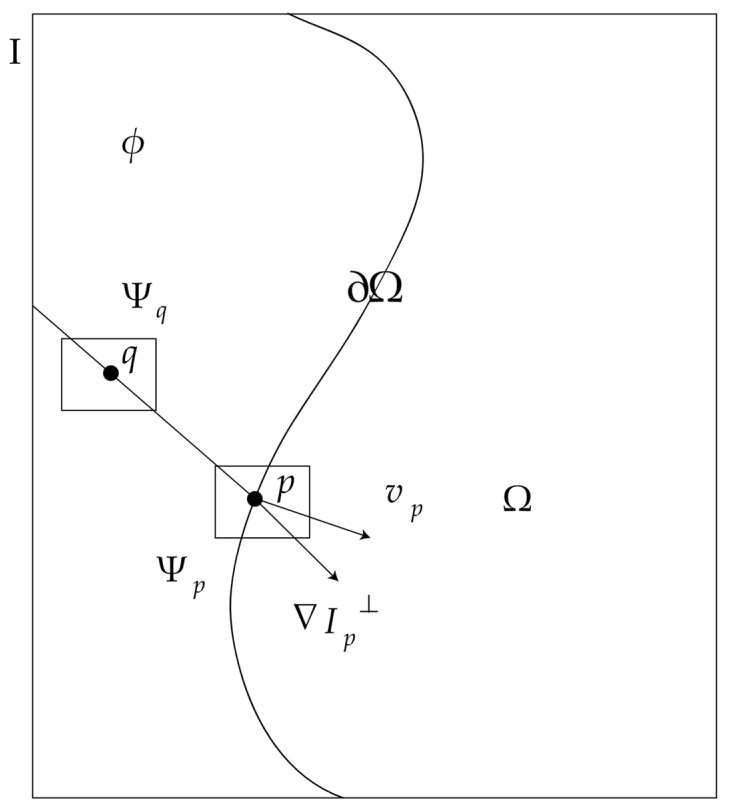
Schematic diagram of block priority calculation.

Figure 1 is a schematic diagram of block priority calculation. I represents the whole image; Ω is the hole pixel area; ∂Ω is the edge contour of the image hole area; *Φ* is the known pixel area (*Φ* = I − Ω); Ψ*_p_* is the broken block (size 3 × 3), whose central pixel is *p*; Ψ*_q_* is the sample block (size 3 × 3), whose central pixel is *q*; and *v_p_* is the normal vector of the center pixel *p* of the broken block, whose vertical direction ∇*I_p_*^⊥^ is the direction where the gray value changes the least (the direction of the isointensity line). Taking the edge point *p* as the central pixel and Ψ*_p_* as the broken block, the priority calculation criterion is defined as follows:

(1) P(p)=C(p)D(p)

where *C(p)* is the confidence term and *D(p)* is the data item:

(2) $$C\left(p\right)=\frac{\sum_{q\in \Psi_{p}\cap \phi }C\left(q\right)}{\left|\Psi_{p}\right|},$$

(3) $$D\left(p\right)=\frac{\left|\nabla I_{p}^{⊥}\cdot v_{p}\right|}{\alpha }$$

where *C*(*q*) is the confidence term of the *q* pixel in the broken block Ψ*p*. During initialization, the function *C*(*p*) is set to *C*(*p*) = 0 ∀*p* ∈ Ω, and *C*(*p*) = 1 ∀*p* ∈ Φ; |Ψ*p*| represents the size of the broken block Ψ*p* (the sum of the number of pixels), and *α* is the normalization factor.

According to *P*(*p*) = *C*(*p*)*D*(*p*), the priority weights of all the broken blocks centered on the pixels in the broken edge area of the image were calculated. The priority weights of the broken blocks could be compared. Therefore, the broken blocks with the maximum priority could be determined and recorded as $\psi_{\hat{p}}$, which is the blocks to be repaired.

### 2.2. Object-Oriented RGB Image Segmentation

The aim of the object-oriented method is to extract the image region, or image object, which is consistent with the real-world object in shape and classification. Compared with traditional image processing and analysis methods, the basic processing unit of the object-oriented image analysis is not a single pixel but the image object extracted after image segmentation. Its biggest feature is that it can generate a lot of new information based on image objects. Compared with a single pixel, homogeneous image objects provide not only hue (spectral) features but also semantic information, such as shape, texture, topology, context, and scale-related features. Using this kind of information, the classification and recognition of each target in the image can be more detailed and accurate. The basic objective of the object-oriented image analysis and application is to extract the image object; that is, the segmentation program should be able to extract the image area (image object), which is consistent with the real-world object in shape and area.

Objects of different sizes and spatial structures of different levels in an image need to be reflected at different scales. One method of extracting the image area is multiscale segmentation, which segments images at different scales so that the information of the object and spatial structure characteristics at different scales in images can be represented by the segmentation results at different scales. Therefore, the goal of the multiscale segmentation algorithm is to divide the image into highly homogeneous, interconnected, and different image regions corresponding to the object or spatial structure features of interest at the corresponding scales.

Image segmentation is essentially a process of dividing a digital image of an *M × N* array into several non-overlapping areas. In the process of object-oriented image segmentation, two algorithms are used: the mean-shift algorithm proposed by Comaniciu and Meer [44], which is used to divide the RGB image into several small regions; and the full lambda schedule algorithm proposed by Robinson et al. [45], which is used to merge small regions with spectral and texture features to avoid over segmentation of texture regions.

#### 2.2.1. Mean-Shift Algorithm

The mean-shift algorithm is a method to obtain a local maximum in densely distributed data (such as image data). It is a nonparametric iterative method to achieve robust data clustering. We use it to estimate the densest region in the multi-dimensional feature space. Different from the traditional clustering methods based on K-means, it does not need to make embedded assumptions about the shape of distribution or the number of clusters. Its density estimation process is based on the Parzen-window [46] technology.

Given n data points, $x_{i}\in R^{d}$, i=1,2,…,n, where d is the feature-space dimension, the multi-variate kernel density at point x can be estimated by a spherical symmetric kernel K(x). The formula is as follows:

(4) $$f_{k}=\frac{1}{nh^{d}}\sum_{i=1}^{n}K\left(\frac{x-x_{i}}{h}\right),$$

where h is the bandwidth parameter and the radius of the kernel is defined. The kernel can be represented by its contour:

(5) $$K\left(x\right)=c_{k,d}k\left(∥x{∥}^{2}\right)>0,\;∥x∥\leq 1,$$

where $c_{k,d}$ is a normalized constant, which ensures K(x) integration with 1. In the feature space, the modes of underlying density f(x) are located among the zeros of the gradient ∇f(x)=0. The gradient of the density estimator (4) can be calculated with the kernel $G\left(x\right)=c_{g,d}g\left(∥x{∥}^{2}\right)$; the profile is defined as $g\left(x\right)=-k^{\prime }\left(x\right)$ with the assumption that the derivative of k(x) exists. The following equation can be obtained:

(6) $$\nabla f\left(x\right)=\frac{2c_{k,d}}{nh^{d+2}}\left[\sum_{i=1}^{n}g\left({∥\frac{x-x_{i}}{h}∥}^{2}\right)\right]\left[\frac{\sum_{i=1}^{n}x_{i}g\left({∥\frac{x-x_{i}}{h}∥}^{2}\right)}{\sum_{i=1}^{n}g\left({∥\frac{x-x_{i}}{h}∥}^{2}\right)}-x\right].$$

The first term of Equation (6) is proportional to the density estimate at x. The second term is called the mean-shift vector, which is proportional to the estimated value of the normalized density gradient at the x point obtained with the kernel K. Therefore, it points in the direction of the fastest increase in density. The expression of the mean shift vector allows us to define an iterative process:

(7) $$y_{t+1}=\frac{\sum_{i=1}^{n}x_{i}g\left({∥\frac{x-x_{i}}{h}∥}^{2}\right)}{\sum_{i=1}^{n}g\left({∥\frac{x-x_{i}}{h}∥}^{2}\right)}-x,\;t=1,2,....,$$

where $y_{t+1}$ is the weighted average value at $y_{t}$ calculated by the kernel G. The mean-shift process of a given point $x_{i}$ is as follows: Initialize $y_{1}=x_{i}$ and apply the recursive expression (7) until convergence, that is, $∥y_{t+1}-y_{t}∥<\varepsilon$, where ε is the threshold. In the iterative process, the vector $y_{t+1}$ points to the corresponding node along a smooth trajectory.

#### 2.2.2. Full Lambda-Schedule Algorithm

The full lambda-schedule algorithm iteratively merges adjacent small objects in the RGB image based on spectral and spatial information. When the algorithm finds two adjacent small blocks i and j, they are merged until the merging cost is greater than a given threshold lambda value $\lambda_{stop}$:

(8) $$t_{i,j}=\frac{\frac{\left|O_{i}\right|\times \left|O_{j}\right|}{\left|O_{i}\right|+\left|O_{j}\right|}{∥u_{i}-u_{j}∥}^{2}}{l\left(\partial \left(O_{i},O_{j}\right)\right)},$$

where $O_{i}$ is object i of the RGB image, $\left|O_{i}\right|$ is its area, $u_{i}$ is the average value in object i, ${∥u_{i}-u_{j}∥}^{2}$ is the Euclidean distance between the spectral values of $O_{i}$ and $O_{j}$. $l\left(\partial \left(O_{i},O_{j}\right)\right)$ is the length of the common boundary of $O_{i}$ and $O_{j}$.

In general, the steps of the full lambda-schedule algorithm are as follows:

Step 1: Take the image objects generated by Section 2.2.1 as the initial trivial segmentation.

Step 2: Search for the pair $\left(O_{i},O_{j}\right)$ that has the smallest $t_{i,j}$ of all the neighboring pairs of objects.

Step 3: Merge the objects $O_{i}$ and $O_{j}$ to form a new object $O_{ij}$.

Step 4: Repeat Steps 2–3 until $t_{i,j}>\lambda_{stop}$ for all pairs of neighboring $\left(O_{i},O_{j}\right)$.

### 2.3. Obtain Best Sample Block

The best sample block in the known pixel area of the image was identified and recorded as $\psi_{\hat{q}}$. An object difference was used to evaluate the similarity between the repairing block $\psi_{\hat{p}}$ and sample block $\psi_{q}$.

(9) $$\psi_{\hat{q}}=\mathrm{argmin}dis\left(\psi_{\hat{p}},\psi_{q}\right),$$

(10) $$\begin{array}{c} dis\left(\psi_{\hat{p}},\psi_{q}\right)=\sum_{i=1}^{n}OD_{i},\\ OD_{i}=\{\begin{array}{c} 0\;,\;O_{\left(x_{i},y_{i}\right)}\neq O_{\left(x_{i}^{\prime },y_{i}^{\prime }\right)}^{\prime }\;\\ 1\;,\;O_{\left(x_{i},y_{i}\right)}\neq O_{\left(x_{i}^{\prime },y_{i}^{\prime }\right)}^{\prime }\; \end{array}\;, \end{array}$$

where $dis\left(\psi_{\hat{p}},\psi_{q}\right)$ is the number of the pixels belonging to different objects in the block to be repaired and in the sample block in the RGB image. This is called object difference. $OD_{i}$ is a number indicating whether the *i*th pixel belongs to the same object in the block to be repaired and in the sample block. $O_{\left(x_{i},y_{i}\right)}$ represents the object that the *i*th pixel in the repairing block $\left(x_{i},y_{i}\right)$ belongs to in the RGB image. $O_{\left(x_{i}^{\prime },y_{i}^{\prime }\right)}^{\prime }$ represents the object that the *i*th pixel in the sample block $\left(x_{i}^{\prime },y_{i}^{\prime }\right)$ belongs to in the RGB image. If the *i*th pixel in the two blocks belongs to the same object in the RGB image, $OD_{i}$ is 0. Otherwise, $OD_{i}$ is 1. *n* is the sum of the number of pixels in the blocks.

### 2.4. Optimize Confidence

After the best sample block $\psi_{\hat{q}}$ is obtained by the aforementioned formula, the pixels in the best sample block were filled into the broken area of the block to be repaired, and the broken edge area of the image was updated. Moreover, the pixel confidence items in the broken area of the block to be repaired were updated

(11) $$C\left(p\right)=C\left(\hat{p}\right)\;\forall p\in \psi_{\hat{p}}\cap \Omega ,$$

where $C\left(\hat{p}\right)$ represents the confidence item $\psi_{\hat{p}}$ value calculated by Formula (2) for the block to be repaired, and C(p) represents the confidence term value of the pixel filled in the broken area of the block with the maximum priority. The aforementioned steps were repeated until the area Ω to be repaired is empty.

### 2.5. Algorithm Flow

For depth image repair, an RGB image was used to search for the best matching block in the depth image. We first search for the block to be repaired having maximum priority, then we segment the RGB image and search for the best sample block with the minimum object difference. Subsequently, we fill the holes, update the broken boundary and optimize confidence. The whole process continues until the extent of the broken block is empty. In general, the main steps of this algorithm are as follows:

Step 1: Set the broken area Ω and boundary area *∂*Ω, and determine the template size of image restoration.

Step 2: According to the priority formula, the broken block with the maximum priority on the edge *∂*Ω is obtained and recorded as the block to be repaired.

Step 3: The RGB image is segmented by a multi-scale object-oriented image, and the small-scale objects are merged.

Step 4: According to the block matching criterion, the best sample block is determined. The object difference is used to search for the best sample block. 

Step 5: Using the best sample block to fill the broken area of the block to be repaired, update the broken boundary, and update the confidence item value of the filled pixel.

Step 6: Check whether the broken area Ω = *∅* is true. If the broken image is completely repaired, then exit the process; otherwise, continue to Step 1.

**Figure 2:**
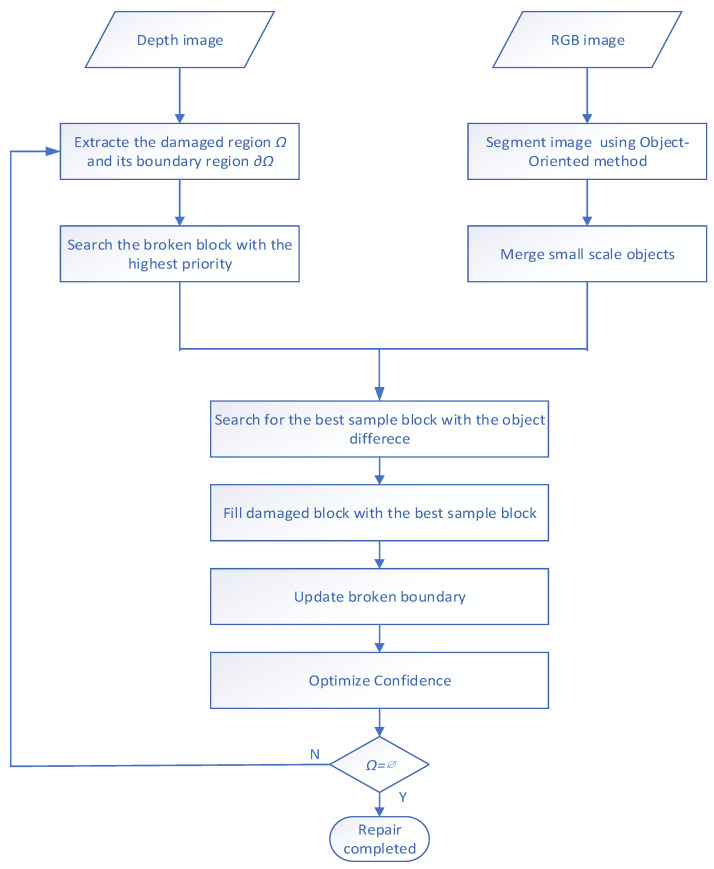
Flow chart of depth image texture synthesis repair.

Figure 2 is the flow chart of the algorithm.

## 3. Experiment

In this section, we introduce the experimental environments and datasets we used, present the depth repair results of the proposed method as well as both the Criminisi and ASB-LS methods. We then draw a comparison among them and numerically evaluate the three methods.

### 3.1. Experimental Environments and Datasets

The algorithms were launched on a computer with an Intel i5 2.50 GHz CPU, 8 GB RAM, and Windows 10 operating system. The codes were written in the MATLAB language.

To verify the effectiveness of the proposed algorithm, the experiments were performed on the RealSense D435i depth images of several public datasets and compared with the algorithms of Criminisi and adaptive sample block and local search (ASB–LS). OpenLORIS-Scene datasets [47], created by researchers at the Inter Research Center and Tsinghua University, have RGB and depth images collected by RealSense D435i with a resolution of 848 × 480 and data from an inertial measurement unit, a fish-eye camera, and a wheel odometer. The data of each sensor were collected at the same time. This study used the RGB and depth image data. The depth image was processed and aligned with the RGB image. We synthesized the ground truths through artificial restoration, referencing information from RGB images. Datasets of four scenes were used: cafe, corridor, home and office environments.

For the quantitative evaluation of the experimental results, we manually filled the holes and constructed ground-truth images from 50 randomly selected depth images of the four aforementioned scenes.

### 3.2. Experimental Results and Comparison

**Figure 3:**
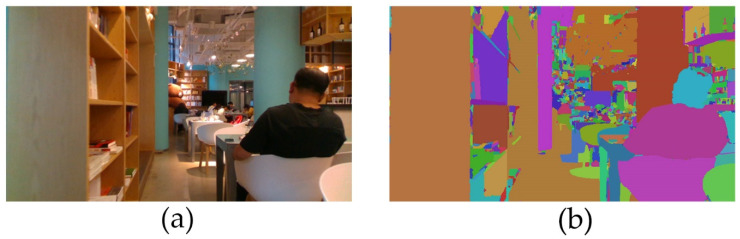
OpenLORIS-Scene Datasets: cafe scene: (**a**) RGB image; (**b**) segmentation of the RGB image.

The RGB image was segmented through the multi-scale object-oriented method. For the cafe scene, the original RGB image is shown in Figure 3a; the result of the multi-scale object-oriented segmentation is shown in Figure 3b. The image was randomly colored according to different objects.

The bookshelf, table, chair, human head, and body in the foreground were divided into different objects based upon heterogeneity. The pillars, walls, chandeliers, monitor, and chairs in the foreground were well-segmented; the other small objects in the foreground were divided into a large number of small pieces.

**Figure 4:**
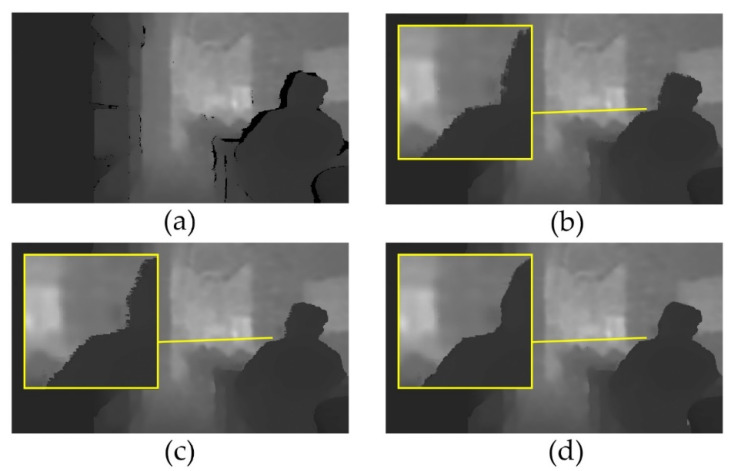
Region filling results of the cafe scene: (**a**) raw depth image; (**b**) Criminisi method; (**c**) ASB–LS method; (**d**) proposed method.

The raw depth image and region filling results of the Criminisi method, ASB–LS method, and the proposed method are shown in Figure 4. In the raw depth image of the cafe scene, a small area of data was missed in the bookshelf part and foreground. Moreover, there were large holes at the edge of the desk, around the table, and at the edge of the human. For small holes, the Criminisi, ASB–LS, and the proposed method had a good filling effect. However, in the filling of large holes, the effects of each method showed obvious differences. The edge around the table and the desk in the Criminisi method were deformed, whereas those of ASB–LS and the proposed method were not. For the filling results of the human edge hole, as shown in the enlarged areas in the figure, the edge of the human was relatively disordered, looked rough; the blocky effect was obvious in the Criminisi method result. The block effect in the ASB–LS method result was significantly reduced, but there were a lot of burrs, mainly because the objects on both sides (foreground and background) were not distinguished during contour matching, and pixel blocks on both sides were filled in the holes, so the pixel values at this place were in a jumping distribution. In the proposed method result, because of the restriction condition that the pixel block is in the same object, the pixel value did not jump, the burr was less, and the edge was smoother.

**Figure 5:**
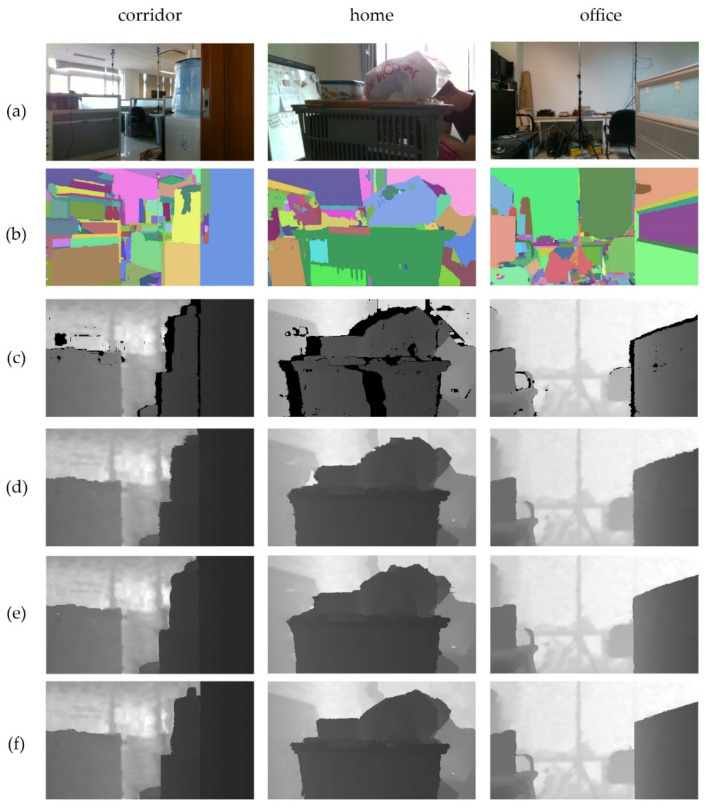
Region filling results of the other scenes: (**a**) RGB image; (**b**) segmentation of the RGB image; (**c**) raw depth image; (**d**) Criminisi method; (**e**) ASB–LS method; (**f**) proposed method.

The RGB images, segmentations, raw depth image, and region filling results of the Criminisi method, ASB–LS method and proposed method of another three scenes are shown in Figure 5.

In the corridor scene, there was a lack of depth data at the edges of the door, the paper cup, the bucket and the drinking fountain. Evidently, there was a gap between the paper cup and the door. The Crimisini and ASB–LS method used the paper cup depth value to fill the gap. On the contrary, because the proposed method limited the gap to find a matching block in its own object, it filled the hole with the depth value of the background. Furthermore, it can be seen that the shape of the paper cup after repairing is consistent with that in the RGB image. For the holes at the edge of the bucket, the Crimisini result had a messy bucket edge and the ASB–LS result was curvilinear, which was inconsistent with the real scene. The shape of the bucket using the proposed method was consistent with that of the RGB image. The hole inside the bucket edge was filled into the depth value of the bucket, and the hole outside the bucket edge was filled into the depth value of the desk or background. For the water dispenser, the proposed method was more accurate than the other two methods. The edge of the repaired water dispenser was more consistent with that of the RGB image.

In the home scene, large holes of deep data appeared around and in the middle of the human, the plastic bag, the lunch box, and the shopping basket. The hole beside the human was repaired well by the three methods, but there was a wrong filling in the ASB–LS results. For the hole above the plastic bag, some parts of the results of the first two methods were filled with the depth value of the plastic bag; the other part was filled with the depth value of the background, and the edge presented an irregular shape. In the proposed result, the depth value of the plastic bag was filled in the area of the plastic bag and the depth value of the background was filled outside the area; hence, the edge presented a shape consistent with the plastic bag in the real scene. For the filling of the left hole in the lunch box, the first two methods not only used the depth value of the whiteboard, but also the depth value of the background to fill in, which is a wrong filling. The hole filling of the first two methods was not based on the area of the two objects. The proposed method filled the depth values of the lunch box or shopping basket to the pixels that belong to their area, and filled the depth values of the adjacent object (white board) to the pixels that were not in the area.

In the office scene, there were holes of depth image at the edge of the computer desk and the edge of the office table. In the left half of the image, the Crimisini and ASB–LS methods filled holes with depth values from other objects; hence, the armrest of the computer desk was broken. In the proposed method, the armrest is continuous and did not break because only the depth data in the computer desk object is used to fill the hole. On the right half of the image, the Crimisini and ASB–LS methods repaired the hole on the edge of the office, and the ASB–LS result was less granular than the Crimisini method. However, in this case, the pixels near the office table were wrongly filled with the depth value of the office table. In fact, the correct depth value should be consistent with the background or the chair next to the office table. In the proposed method, the hole pixels in the area of the background object are filled with the depth value of the background, and those in the area of the chair object were filled with the depth value of the chair. The hole pixels that are not in the area of the office table object were not wrongly filled with the depth value of the office table.

### 3.3. Numerical Evaluations

To numerically evaluate the hole-filling methods, the root mean square error (RMSE), peak signal-to-noise ratio (PSNR), and structural similarity index measure (SSIM) from the hole-filled depth images and ground-truth images were calculated. The filled hole areas were defined as a set *S* to be used for our comparison with the ground truth. The RMSE is defined as follows:

(12) $$\mathrm{RMSE}=\sqrt{\frac{1}{n}\sum_{\left(x,y\right)\in S}{\left(depth_{G}\left(x,y\right)-depth_{H}\left(x,y\right)\right)}^{2}},$$

where *n* is the number of pixels in *S*, (*x,y*) are their coordinates, $depth_{G}\left(x,y\right)$ is the ground-truth depth value, and $depth_{H}\left(x,y\right)$ is depth value of the hole-filled depth image.

The PSNR is defined as follows:

(13) $$\mathrm{PSNR}=10\log_{10}\left(\frac{\max{\left(d_{i}\right)}^{2}}{\mathrm{MSE}}\right),$$

where $\max\left(d_{i}\right)$ is the maximum depth value of the hole-filled depth image. MSE is the mean square error between a hole-filled depth image and ground-truth image, square of RMSE. 

As another evaluation index of depth image hole-filling quality, SSIM measures the structural similarity of the image quality between the ground-truth and hole-filled depth image. It is defined as follows:

(14) $$\mathrm{SSIM}\;=\;\frac{\left(2\mu_{x}\mu_{y}+C_{1}\right)\left(2\sigma_{xy}+C_{2}\right)}{\left(\mu_{x}^{2}+\mu_{y}^{2}+C_{1}\right)\left(\sigma_{x}^{2}+\sigma_{y}^{2}+C_{2}\right)},$$

where $\mu_{x}$ and $\mu_{y}$ are the means of the depth value $depth_{G}$ and $depth_{H}$, $\sigma_{x}^{2}$ is the variance of $depth_{G}$, $\sigma_{y}^{2}$ is the variance of $depth_{H}$, and $\sigma_{xy}$ is the covariance between the depth value $depth_{G}$ and $depth_{H}$.

The RMSE, PSNR, and SSIM of the hole-filled image of the four scenes for the Criminisi, ASB–LS, and the proposed method are shown in Table 1. 

For the cafe scene, the ASB-LS method had smaller RMSE and larger PSNR than the Criminisi method, indicating that it was closer to ground truth. The RMSE of the proposed method is smaller than that of the former two, and PSNR and SSIM are larger than the former two, indicating that it was the closest to ground truth among the three methods. In other words, it has the best cavity repair effect. For the corridor scene and the home scene, the RMSE of the Criminisi method was smaller than that of the ASB-LS method, PSNR is larger, and SSIM is larger. The former was closer to ground truth than the latter. The proposed method had smaller RMSE, larger PSNR and larger SSIM than the Criminisi method, which was the closest to ground truth among the three methods. For the office scene also, the proposed method had the best evaluation index, which was closest to the ground truth. The same was true for 50 randomly selected depth images after repair.

## 4. Conclusions

Owing to the mechanism of hardware systems, there are holes in the depth image collected by the RealSense D435i depth camera. We used texture synthesis to repair the depth image. Different from the previous Criminisi and ASB-LS methods, we used the RGB images which were collected simultaneously with the depth images in this study. After the RGB image is segmented by an object-oriented method, we define an object difference parameter as the principle to find the best sample block. In RGB image segmentation results, the same object may be divided into several different objects, which will not affect the depth repair, because the depth value of the same object is the same or close. This way to find the best sample block ensures that the blocks belonging to other objects will not be filled into the blocks to be repaired, so as to improve the accuracy of depth image repair.

We compared the proposed method with those of two other scholars, the Criminisi and ASB-LS methods. When the Criminisi method is used to repair large areas of cavity, the visual performance of the edge is rough and the graininess is obvious. Because the pixels in the cavity are filled with pixels close to it, some of the filled depth values are wrong. The ASB-LS method eliminates the grain sense at the edge, but there are some burrs and wrong filling. In the optimal matching process, the proposed method adds the object limitation generated by multi-scale object-oriented segmentation, avoids error filling, makes the edge consistent with the edge of RGB image, and improves the visual effect. After the numerical evaluation of these three methods, we can see that for some scenarios, the ASB-LS method is better than the Criminisi method in numerical evaluation, whereas in other scenarios it is the opposite. But in all scenarios, the proposed method has better numerical evaluation than the other two methods. It shows that the proposed method has the highest repair accuracy. In addition, we randomly selected 50 depth images, synthesized the ground truth through artificial restoration, and then used three methods to repair, which also showed the same conclusion.

After the depth image has been repaired, we can apply the depth image to some applications in future work to better play its role. For example, in the 3D model reconstruction of RGB-D images, faults can be avoided. In the RGB-D slam system, it can solve the pose and map better in real time.

## Figures and Tables

**Table 1:** Method comparison based on the ground-truth image with root mean square error (RMSE), peak signal-to-noise ratio (PSNR), and structural similarity index measure (SSIM).

Image of Scenes	Criminisi Method [33]			ASB–LS Method [35]			Proposed Method		
	RMSE	PSNR	SSIM	RMSE	PSNR	SSIM	RMSE	PSNR	SSIM
Cafe	3.287	37.796	0.996	3.085	38.347	0.996	2.691	39.534	0.997
Corridor	20.589	21.858	0.959	21.501	21.482	0.953	18.630	22.726	0.969
Home	22.606	21.047	0.942	22.753	20.990	0.941	13.810	25.327	0.978
Office	12.800	25.987	0.987	13.484	25.535	0.983	7.421	30.721	0.995
Average of 50 images	14.123	26.162	0.952	14.396	26.180	0.953	12.127	27.766	0.959
